# Determination and Removal of Selected Pharmaceuticals and Total Organic Carbon from Surface Water by Aluminum Chlorohydrate Coagulant

**DOI:** 10.3390/molecules27175740

**Published:** 2022-09-05

**Authors:** Joanna Kuc, Maciej Thomas, Iwona Grochowalska, Rafał Kulczyk, Gabriela Mikosz, Fabian Mrózek, Dagmara Janik, Justyna Korta, Karolina Cwynar

**Affiliations:** 1 Faculty of Chemical Engineering and Technology, Cracow University of Technology, Warszawska 24, 31-155 Cracow, Poland; 2 Faculty of Environmental Engineering and Energy, Cracow University of Technology, Warszawska 24, 31-155 Cracow, Poland; 3 Faculty of Natural Sciences, Jan Kochanowski University of Kielce, Żeromskiego 5, 25-369 Kielce, Poland; 4 Student Scientific Association of Environment and Food Analytics, Faculty of Chemical Engineering and Technology, Cracow University of Technology, Warszawska 24, 31-155 Cracow, Poland

**Keywords:** pharmaceuticals, coagulation, response surface methodology, erythromycin, fluoxetine, amoxicillin, colistin, ethynylestradiol, diclofenac, RP-HPLC-ESI-MS/MS

## Abstract

In the present research, the removal of Total Organic Carbon (TOC) and erythromycin (ERY), fluoxetine (FLX), amoxicillin (AMO), colistin (COL), ethynylestradiol (EE), and diclofenac (DIC) from surface water by coagulation is studied. The concentration of selected pharmaceuticals in 24 surface water samples originating from some rivers located in Lesser Poland Voivodeship and Silesia Voivodeship, Poland, was determined. The removal of TOC and pharmaceuticals was carried out using the application of Design of Experiments (DOE), Response Surface Methodology (RSM), and by addition of aluminum chlorohydrate (ACH) as a coagulant. The study found that the concentration ranges of ERY, FLX, AMO, COL, EE, and DIC in analyzed water samples were 7.58–412.32, 1.21–72.52, 1.22–68.55, 1.28–32.01, 5.36–45.56, 2.20–182.22 ng/L, respectively. In some cases, concentrations lower than 1 ng/L were determined. In optimal conditions of coagulation process of spiked surface water (pH = 6.5 ± 0.1, ACH dose = 0.35 mL/L, Time = 30 min; R^2^ = 0.8799, R^2^_adj_ = 0.7998), the concentration of TOC, ERY, FLX, AMO, COL, EE, and DIC was decreased by 88.7, 36.4, 24.7, 29.0, 25.5, 35.4, 30.4%, respectively. Simultaneously, turbidity, color, Total Suspended Solids (TSS), Chemical Oxygen Demand (COD), Total Nitrogen (Total N), and Ammonium-Nitrogen (N-NH_4_) were decreased by 96.2%, >98.0%, 97.8%, 70.0%, 88.7%, 37.5%, respectively. These findings suggest that ACH may be an optional reagent to remove studied pharmaceuticals from contaminated water.

## 1. Introduction

The societal demographic situation of the European Union (EU), which currently comprises 27 democratic countries, is changing very dynamically compared with other parts of the world [1]. The increase in life expectancy over the last century depends on a number of factors, including the reduction of infant mortality through improved lifestyle, economic development, increased social awareness, accident prevention, as well as advances in healthcare, medicine, and pharmacology [2]. Demographic evolution is undoubtedly one of the main factors influencing the dynamics of the pharmaceutical industry’s development and the growing consumption of drugs in society, as well as their use in veterinary medicine, which is inevitably associated with related environmental pollutants [3]. Numerous studies have confirmed the direct impact of individual pharmaceutical preparations on flora and fauna [4]. The issue of pharmaceutical residues remains critical from the point of view of long-term exposure causing negative effects for humans, animals, and the environment. An example is the presence of diclofenac in aquatic ecosystems and its harmful effect on the reproduction of freshwater rowers (*Daphnia magna* and *Moina macrocopa*) [5], pathological changes in the kidneys, hyperplasia, and fusion of the villi in the intestine (*Oncorhynchus mykiss*) [6] or a radical decline in the population of the Bengal vulture (*Gyps bengalensis*) feeding on carrion treated with diclofenac [7,8]. Other examples show that 17α-ethynylestradiol prevents the natural male-to-female sex change in gilthead seabream (*Sparus aurata* L.) [9]. Fluoxetine residues found in many freshwater environments significantly influence mating behavior in male fish [10]. It is similar to the residues of antibiotics, which enter rivers and lakes, accumulates in the soil, and adversely affect living organisms.

Determining the scale of the problem of water and soil contamination with pharmaceutical residues is a strategic challenge that the EU has met. The purposefulness of monitoring environmental pollutants from the Pharmaceuticals and Personal Care Products (PPCPs) group is dictated by a preventive approach that proves the growing awareness of the European Commission in the face of the documented and growing threat posed by biologically active compounds [11,12,13].

The durability of pharmaceutical preparations is varied and is mainly due to the chemical structure as well as the nature of the aquatic environment. Some of them will be easily transformed by natural processes, such as biodegradation, photodegradation, or absorption by plants or microorganisms and the related biotransformation. However, contamination with compounds from pharmaceutical groups is defined as “pseudo-persistent,” which results from the fact that their emission is continuous and despite progressing degradation processes, these compounds have a high potential for persistence in the environment, which is exacerbated by the increasing consumption of drugs [14,15,16].

The efficiency of removing pharmaceutical residues depends on the level of treatment, the degree of advancement of the installation, as well as the nature of the substances present in the sewage. Pollutants pass through sewage treatment plants and enter the aquatic ecosystems in varying amounts. On the other hand, non-point sources include pollutants, the location of which is difficult to identify, such as runoff from agricultural soils, urban runoff, or unidentified leaks from wastewater treatment systems and plants [17]. Pharmaceutical-related emission sources are classified in two ways: point sources and area/diffusion (non-point) sources. The first mentioned source refers to single, easy-to-locate sources of pollution, such as domestic wastewater discharges, domestic solid wastes, wastewater from the pharmaceutical industry, wastewater, hospital wastes (biomedical wastes), and wastewater treatment plants. Another point source is treated/untreated sewage from wastewater treatment plants. The “non-point” sources are defined as any other pollution sources that do not meet the point source definition. Unfortunately, sewage treatment plants do not completely eliminate pharmaceutical pollutants [18,19].

Various methods are used to remove pharmaceuticals from water and wastewater, e.g., physical treatment (primary treatment), aerobic and anaerobic processes (biological treatment), adsorption, membrane technologies (tertiary treatment), Fenton process, O_3_/H_2_O_2_ treatment, photocatalytic processes, ultrasound irradiation, wet air oxidation, etc., (Advanced Oxidation Processes, AOPs) and hybrid technologies [20]. Chemical coagulation is one of the methods used to remove organic pollutants, e.g., pharmaceuticals, from water. The application of alum (Al_2_(SO_4_)_3_ at pH 6 and ferric sulphate (Fe_2_(SO_4_)_3_) at pH 4.5 indicated that the removal rate for diclofenac, ibuprofen, bezafibrate was 77%, 50%, and 36%, respectively [21]. On the one hand, the application of Al_2_(SO_4_)_3_ in the presence of humic acid resulted in an increase in the removal of naproxen and diclofenac (61% and 59%, respectively). On the other hand, the results showed that neither acetaminophen nor carbamazepine was removed effectively (<10%) [22]. The use of a bench-scale system (coagulation, flocculation, sedimentation, filtration, and chlorination) resulted in the removal of caffeine, trovafloxacin mesylate, estradiol, and salicylic acid (charge of pharmaceuticals at pH 8: neutral, zwitterionic, neutral, and negative, respectively) by 3.4–13.0, 21.0–31.0, 6.9–12.0, and 31%–39%, respectively; (Al_2_(SO_4_)_3_ dose 25 mg/L, pH 8, rapid mixing 9.3 min, flocculation time 39.2 min) [23]. In other studies for diclofenac (DCF), naproxen (NPX), and ibuprofen (IBP), a maximum removal rate of 46%, 42%, and 23%, respectively, were obtained [24]. Based on published data, the removal rate for erythromycin (Al_2_(SO_4_)_3_, 78 mg/L, pH 6.8), fluoxetine (Al_2_(SO_4_)_3_, 78 mg/L, pH 6.8), ethynylestradiol (polyaluminum chloride (PACl), 5.4 mg/L, pH 6.8–7.2), and diclofenac (Fe_2_(SO_4_)_3_, 94 mg/L, pH 4.5–4.9) was 33%, 15%, 21% and 8%–77%, respectively [25,26,27]. Literature data indicate that Al_2_(SO_4_)_3_), PACl, Fe_2_(SO_4_)_3,_ and FeCl_3_ are commonly used as coagulants [28,29,30]. Inorganic coagulants have many advantages. They are widely used, easy to apply, relatively cheap, and do not require high operating costs. Aluminum chlorohydrate (ACH) was used for the coagulation of river water [31], textile wastewater [32], and others but was not applied for the removal of ERY, FLX, AMO, COL, EE, and DIC by coagulation.

In this study, the results of the research regarding the content of selected pharmaceuticals in 24 surface water samples originating from some rivers located in Lesser Poland Voivodeship and Silesia Voivodeship, Poland, were presented. In order to investigate the applicability and potential of ACH as a coagulant, it was adopted in the spiked river water purification process. DOE and RSM were used to determine the most favorable conditions (pH, ACH dose, Time) for the coagulation process. Based on the experimental data, a process model was created and experimentally verified. The effectiveness of the coagulant (ACH) under the most favorable conditions was evaluated.

## 2. Results

### 2.1. Occurrence of Diclofenac (DIC), Fluoxetine (FLX), Ethynylestradiol (EE), Erythromycin (ERY), Amoxicillin(AMO), and Colistin (COL) in Surface Water

The standard curve method was used in the quantification of pharmaceuticals. Standard solutions were prepared in the concentration range from 1 to 1000 ng/L. The coefficients of determination (R^2^) were over 0.999 for each of the compounds, which proves the linearity of the method. The limit of quantification (LOQ) was determined from the signal-to-noise ratio (S/N). The LOQ was 1 ng/L for each of the tested compounds.

**Figure 1:**
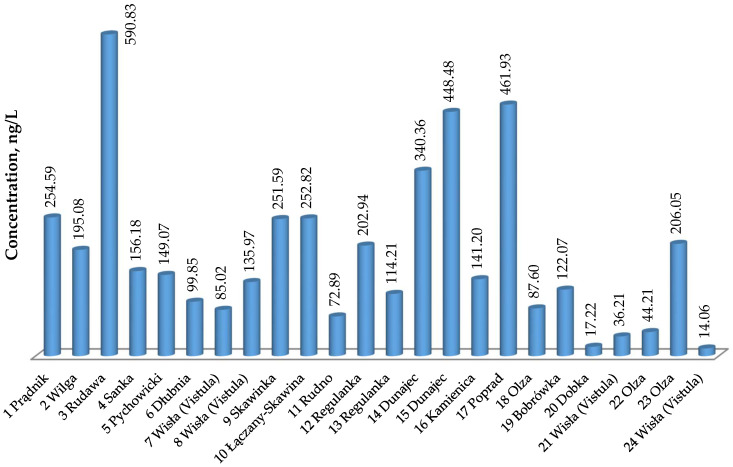
The total content of diclofenac (DIC), fluoxetine (FLX), ethynylestradiol (EE), erythromycin (ERY), amoxicillin (AMO), and colistin (COL) determined in the studied watercourses, ng/L.

Table 1 presents the results of the content of selected pharmaceuticals in samples taken from rivers, streams, and canals in southern and southwestern Poland in 2021. The locations of the tested watercourses and the sampling sites are described in Section 4.2. The overall assessment of the purity of the analyzed watercourses as the sum of determined pharmaceuticals is presented in Figure 1.

### 2.2. Removal of TOC, Diclofenac (DIC), Fluoxetine (FLX), Ethynylestradiol (EE), Erythromycin (ERY), Amoxicillin(AMO), and Colistin (COL) in Surface Water

The research was carried out using river water to which specific amounts of DIC, FLX, EE, ERY, AMO, and COL were added. The analytical tests were carried out in accordance with the methods presented in detail in Section 4.3. Table 2 shows the selected physicochemical parameters of the spiked river water used in the study.

Optimization of the TOC removal process from the spiked river water was performed by designing the experiments using the Central Composite Design (CCD) and Response Surface Methodology (RSM). The criteria for selecting independent parameters and their values, as well as the method of planning the experiments and their way of performing, are presented in Section 4.4. Table 3 depicts the experimental conditions for RSM and the results of the experiments (concentration of Total Organic Carbon, mg/L, and Efficiency of the removal process, %).

The experimental data were statistically analyzed using Statistica 13. Table 4 presents the initial evaluation of the effects and the R^2^ and R^2^_adj_ values assuming the participation of all independent parameters and their possible linear (L) and quadratic (Q) interactions.

Table 5 shows the evaluation of the effects and the change in the R^2^ and R^2^_adj_ values after removing statistically insignificant linear interactions, i.e., pH (L)–ACH dose (L), pH (L)–Time (L), and ACH dose (L)–Time (L).

Table 6 shows the results of verification of the adequacy of the model by ANOVA, and Table 7 presents calculated linear (L) and quadratic (Q) coefficients of the fitted model.

**Figure 2:**
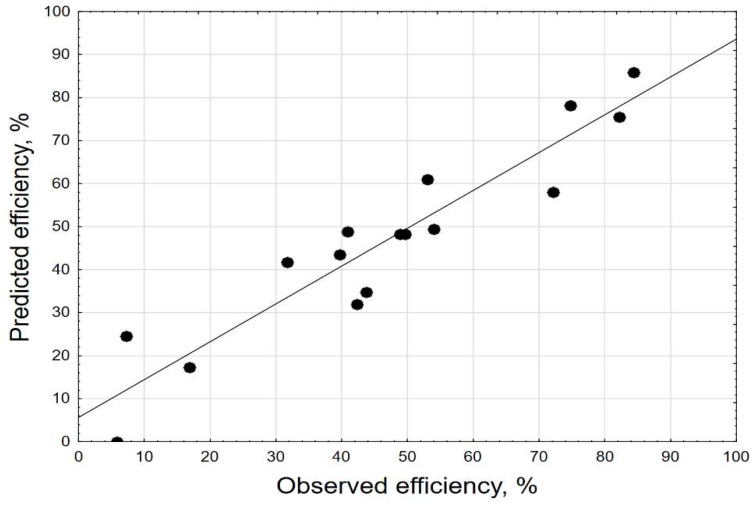
Observed vs. estimated values (Efficiency, %, 3 value, 1 block, 16 experiments, MS = 115.9099, R^2^ = 0.8799, R^2^_adj_ = 0.7998).

**Figure 3:**
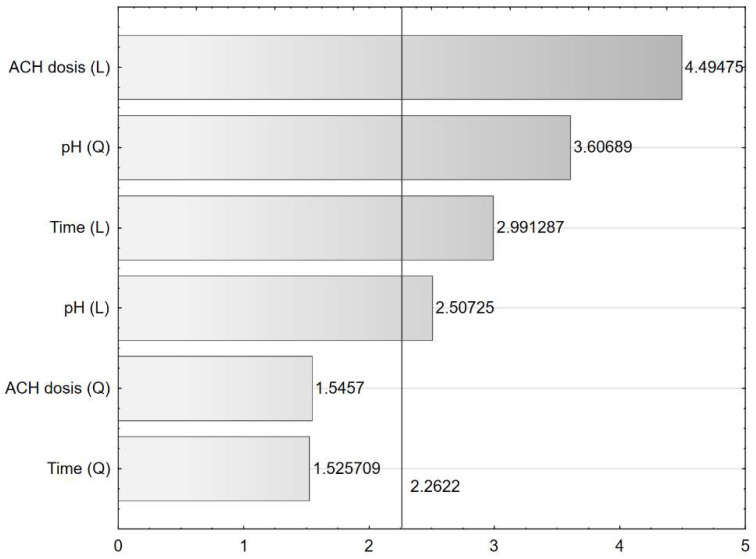
Bar chart of standardised effects (3 value, 1 block, 16 experiments, MS = 115.9099, R^2^ = 0.8799, R^2^_adj_ = 0.7998, L-linear effect, Q-quadratic effect) 2.2622–the absolute value of the standardised effect assessment for *p* = 0.05.

Figure 2 shows the relationship between observed and estimated efficiency from the model, and Figure 3 presents a bar chart of the standardized effects. The 2.2622–value indicated the absolute value of the standardized effect assessment for *p* = 0.05.

**Figure 4:**
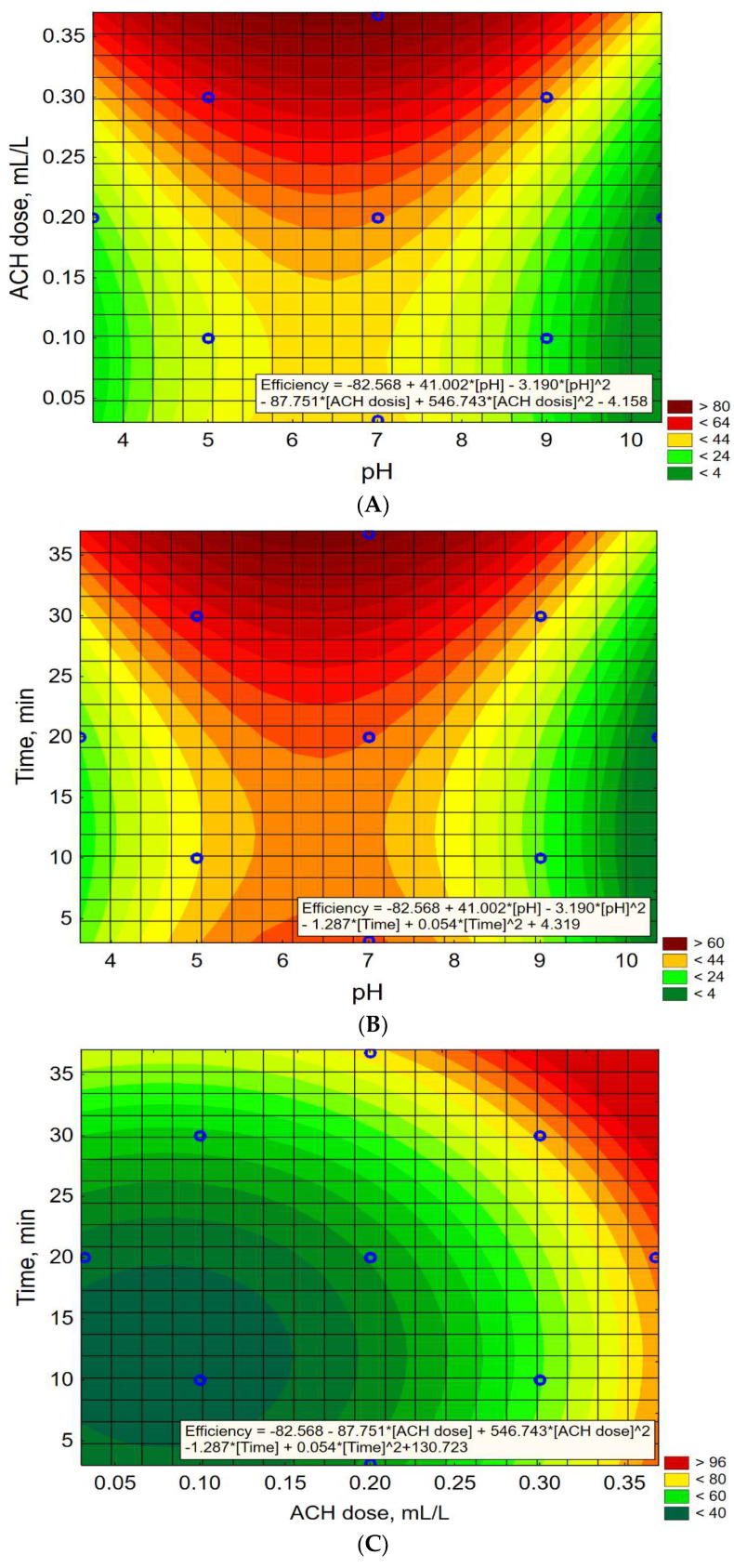
Response surface plots for Efficiency, % with respect to pH and ACH dose (mL/L) for constant Time = 20 min (**A**), pH and Time (min) for constant ACH dose = 0.2 mL/L (**B**), and ACH dose (mL/L) and Time (min) for constant pH =7.0 (**C**).

Figure 4A–C show the changes in the value of the dependent parameter (Efficiency, %) depending on the combination of two independent parameters selected from pH-value, ACH dose, mL, and Time, min. The value of the third independent parameter not shown in the graph is constant.

Table 8 displays the results of experimental model verification. Table 9 shows the changes in selected physicochemical parameters of spiked river water after treatment in optimal conditions based on the model, including TOC and concentration of DIC, FLX, EE, ERY, AMO, and COL.

## 3. Discussion

### 3.1. Occurrence of Diclofenac (DIC), Fluoxetine (FLX), Ethynylestradiol (EE), Erythromycin (ERY), Amoxicillin(AMO), and Colistin (COL) in Surface Water

All results are measurements of instantaneous concentrations and not continuous monitoring of a given watercourse, which could be carried out, for example, with the use of passive samplers with long-term sorption of the analyte. Nevertheless, instantaneous concentration measurements provide information on the leakage of pharmaceutical residues into the aquatic environment in a given area. They can be a valuable source of information in risk assessment.

The total content of tested pharmaceuticals (Figure 1) in the analyzed watercourses indicates that the sample taken from the Rudawa river in Kraków (sample no. 3) was most contaminated. The result was determined by the high content of ERY at the level of 412.32 ± 11.96 ng/L. It is also the highest concentration of all the results obtained. The remaining analytes in this sample have concentration values comparable to the remaining samples. The high concentration of erythromycin in the sample in question may be related to the use of this compound in the treatment of farm animals, the leachate of which may flow into the Rudawa River from its source to its mouth or by deliberate pollution.

The next rivers with a high total content of pharmaceuticals were the Poprad (sample no. 17) and the Dunajec (samples no. 14 and no. 15). The highest concentrations in these samples were observed for DIC and ERY. The Poprad River is a right tributary of the Dunajec in the city of Nowy Sącz. Sample no. 14 was collected within the city, while sample no. 15 was collected within the village of Łącko, near the Nowy Sącz. Pharmaceutical contamination of these rivers may be related to the leachate from households along the course of these rivers as well as problems with wastewater treatment, which has been repeatedly pointed out by the Polish media [33].

The following samples were the cleanest in relation to the content of the tested pharmaceuticals: sample no. 24, taken from the Vistula from an area behind the sewage treatment plant, sample no. 20 from the mountain stream Dobka, flowing to the right into the Vistula within Ustroń, which is a spa town, then sample no. 21 from the municipal water intake point on the Vistula and sample no. 22 from the Olza River in the sparsely populated village of Pogwizdów.

DIC was determined in all tested samples. In samples from the Vistula River collected in Krakow, the DIC concentration values were observed at a level of 75.55 ± 2.12 ng/L (sample no. 7) and 27.05 ± 0.89 ng/L (sample no. 8). A positive aspect is that these values are lower than those recorded by researchers [34] in 2012 in the Vistula (140 ng/L).

The highest concentration value for FLX (72.52 ± 3.77 ng/L) was observed in sample no. 23, collected from the Olza River before the sewage treatment plant, and the lowest concentration (1.22 ± 0.05 ng/L) was obtained in sample no. 21 from the municipal water intake point on the Vistula in Cieszyn Silesia. Within samples from the Vistula in Krakow, FLX levels were determined as 12.26 ± 0.64 ng/L (sample no. 7) and 6.22 ± 0.18 ng/L (sample no. 8). These values are higher than those obtained in the Vistula River flowing through Warsaw in 2014 [35].

When assessing individual measurement values, the physicochemical properties of individual pharmaceuticals and the location of the sampling points should be taken into account. The concentration of individual analytes will be influenced by the location of watercourses in highly populated and urbanized regions, in front of or behind wastewater treatment plants, or on the contrary—in rural, less populated areas but not always connected to wastewater treatment plants. The presence of pharmaceuticals in farmland will also depend on intensified breeding of animals that are treated with antibiotics such as ERY, AMO, and COL. For example, the content of EE (12.59 ± 0.42 ng/L), AMO (35.66 ± 1.78 ng/L), and COL (12.51 ± 0.44 ng/L) in sample no. 18 was significantly higher than in sample no. 22, in which EE was < LOQ, and the concentrations of AMO and COL were almost three times lower (12.45 ± 0.62 ng/L and 4.65 ± 0.16 ng/L, respectively). Both samples were taken from the Olza River, but sampling point 18 was located in the vicinity of two large border cities: Cieszyn and Český Těąín. In contrast, sample 21 was collected in a smaller city, behind the sewage treatment plant. DIC, FLX, and ERY were present at similar concentrations in these samples, but the values were not very high compared with the other samples.

It is also worth paying attention to the results of COL and AMO in samples 19 and 20. In the Bobrówka stream (sample 19), which is a tributary of the Olza River, the highest concentrations of COL and AMO among the tested samples were recorded. The course of the Bobrówka can be observed in a highly agricultural area with many farms breeding cattle and pigs. In addition, not all nearby houses and industrial plants are properly channeled. These results are not surprising because the Bobrówka stream is attributed to the source of domestic sewage runoff from nearby houses, workplaces, and leachate from the surrounding fields and meadows.

The Rudno stream (sample 11) is a good example confirming the dependence of the concentrations of pharmaceuticals on the location of the watercourse. Both the source and the course of the Rudno stream mean that the river meanders through the Tenczyński Landscape Park and the Nature Reserve, passing densely populated areas with intensive animal husbandry, it is not surprising that the concentrations of FLX, ERY, and AMO were <LOQ, and the concentration of COL was determined in trace amounts (2.14 ± 0.08 ng/L). However, this stream partly flows along a 1.5 km section of the village of Przeginia Duchowna, and perhaps the uncontrolled runoff of domestic sewage had an impact on the determination of DIC (38.00 ± 1.19 ng/L) and EE (32.75 ± 1.08 ng/L) in the sample.

When comparing individual rivers and streams with respect to individual pollutants, attention should also be paid to the possibility of the dilution of pollutants in large watercourses, differences in water depth and purity, the size of bottom sediment, or the presence of fauna and flora. Such differences occur between samples no. 12 and 13 from the Regulanka River. Sampling point no. 12 is located within the sparsely populated city center of Alwernia, while sampling point no. 13 is situated outside the city in an unpopulated area. In sample no. 12, taken from the city, all pharmaceuticals tested had higher concentrations than in the sample outside the city, where FLX, EE, and AMO were determined < LOQ. This may indicate the possibility of the accumulation of drugs in the bottom sediment because Regulanka is classified as a meandering river so a large amount of sediment is retained along its course. Additionally, after passing through the city, this river is diluted with treated wastewater from a chemical plant located in Alwernia.

When analyzing samples taken from rivers within Krakow (samples 1–8), it is noticed that in the Vistula and its tributaries, the studied pharmaceuticals are present at comparable levels of concentration. This confirms that pollutants dissolved in water, such as pharmaceuticals, have the ability to accumulate in the bottom sediments of tributaries, as confirmed by other researchers [36,37,38].

### 3.2. Removal of TOC and Diclofenac (DIC), Fluoxetine (FLX), Ethynylestradiol (EE), Erythromycin (ERY), Amoxicillin(AMO), and Colistin (COL) from Spiked Surface Water

The spiked river water used in the study was slightly alkaline (pH = 7.7), and the concentration of TOC and COD responsible for the presence of organic compounds in the tested water was 151 ± 23 mg/L and 676 ± 101 mg O_2_/L. The other parameters of tested water, including the initial concentration of pharmaceuticals, are presented in Table 2. The tests were carried out in accordance with the experimental plan (Table 3) to find the optimal conditions for the coagulation process and TOC removal. TOC is considered one of the most important water quality indicators [39]. The experimental results showed that the lowest efficiency (<8%) was obtained in the experiments numbered 9 and 10. The same ACH dose (0.2 mL/L) and coagulation time (20 min) were used in both experiments. However, in both cases, extreme pH values were used, i.e., pH = 3.6 and pH = 10.4, respectively. In the case of ACH and the tested water used in this study, the results indicated that extreme pH values are not appropriate and will not achieve the maximum efficiency of TOC removal. The highest efficiency (>80%) was achieved in experiments no. 4 and 12, in which pH = 5 and pH = 7 were used. In both cases, 0.3 and 0.37 mL/L of ACH were used, and a reaction time of 30 and 20 min was applied. The analysis of the obtained results suggested that the maximum efficiency of TOC removal can be achieved when the pH value of treated spiked river water is between 5 and 7. Overall, the TOC value varied from 23.6 ± 2.4 to 142.3 ± 14.2 mg/L, and the process efficiency was in the range of 5.8–84.4%.

The obtained experimental results were statistically evaluated. The evaluation of the effects indicated four statistically significant parameters, i.e., constant value, pH (Q), ACH dose (L), and Time (L). As a result of performed analysis, the non-statistically significant linear interactions were identified, i.e., pH (L)–ACH dose (L), pH (L)–Time (L), and ACH dose (L)–Time (L). The coefficient of determination denoted R^2^, is the proportion of the variation in the dependent variable (Efficiency, %) that is predicted by the statistical model. In other words, the R^2^ value reveals what percentage of the variability in the target variable (Efficiency, %) is explained by the regression model. Adjusted R^2^ is a corrected goodness-of-fit (i.e., model accuracy) measure for linear models but adjusts for the number of parameters in a model. Adding more statistically insignificant input parameters to the model causes a decrease in the R^2^_adj_ value while adding more significant parameters causes an increase in the R^2^_adj_ value (R^2^ ≥ R^2^_adj_). R^2^ and R^2^_adj_ were calculated as follows:

(1) $$R^{2}=1-\frac{SS_{residual}}{SS_{model\;}+SS_{residual}}$$

(2) $${R^{2}}_{\mathrm{adj}}=1-\frac{n-1}{n-p}\;(1-R^{2})$$

where *SS* is the sum of the squares, *n* is the number of experiments, and *p* is the number of predictors, not counting the constant value [39,40,41].

The coefficient of determination (R^2^) and the adjusted coefficient of determination (R^2^_adj_) were 0.8977 and 0.7442, respectively (Table 4). After removing the indicated linear interactions from the model, five statistically significant independent parameters were identified, i.e., constant value, pH (L), pH (Q), ACH dose (L), and Time (L). The coefficient of determination (R^2^) and the adjusted coefficient of determination (R^2^_adj_) were 0.8799 and 0.7998, respectively (Table 5). The R^2^ value reveals that 87.99% of the variability in the target variable (Efficiency, %) is explained by the regression model. After removing the indicated linear interactions from the model, the R^2^_adj_ value increased from 0.7442 to 0.7998. The R^2^_adj_ value increased because the newly added predictor (pH (L)) improved the model’s predicting power. It is clear because adding independent and not statistically relevant predictors to the regression model results in a decrease in the R^2^_adj_ value. Generally, it can be assumed that if 0 < R^2^ ≤ 0.5, the fit of the model is unsatisfactory if 0.5 < R^2^ ≤ 0.6, the fit of the model is poor if 0.6 < R ^2^ ≤ 0.8, the fit of the model is satisfactory if 0.8 < R^2^ ≤ 0.9, the fit of the model is good if 0.9 < R^2^ ≤ 1, the fit of the model is very good. For instance, in other studies, the following values of R^2^ and R^2^_adj_ were obtained and evaluated: 0.9683 and 0.9208 (very good fit) for removal of cadmium from polluted water [42], 0.8799 and 0.7999 (good fit) for treatment of textile wastewater using potassium ferrate and Fe(III)/H_2_O_2_ [41], 0.9307 and 0.8845 (very good fit) for removal of heavy metal ions industrial wastewater by sodium trithiocarbonate [43], 0.9161 and 0.8961 (very good fit) for removal of chelated copper ions from industrial wastewater [40], 0.9143 and 0.8530 (very good fit) for TOC removal by alum in the coagulation process [39]. The R^2^ and R^2^_adj_ obtained in this study (0.8799 and 0.7998, respectively), indicating a good fit of the model to experimental data.

The adequacy of the model coefficients was verified by means of ANOVA. The conducted analysis showed (Table 6) 4 statistically significant input parameters, i.e., pH (L), pH (Q), ACH dose (L), and Time (L).

The calculated intercept, linear (L), and quadratic (Q) coefficients of the fitted model are listed in Table 7. The regression model enables the modeling of the response as a mathematical function of several continuous factors, and good estimates of the model parameters are necessary. Each response (e.g., Efficiency, %) can be expressed by a mathematical equation that describes the response surface. The following second-order polynomial equation is suitable for a mathematical description of the response function:

(3) $$\mathrm{Efficiency},\;\%=\beta_{0}+\sum_{i=1}^{k}\beta_{i}x_{i}+\sum_{i=1}^{k}\sum_{j=i+1}^{k}\beta_{ij}x_{i}x_{j}+\beta_{1}x_{1}+\sum_{i=1}^{k}\beta_{ii}x_{i}^{2}$$

where Efficiency, % is the dependent parameter, *β*_0_ is the constant coefficient, *β_i_*_,_ *β_ij,_* and *β_ii_* are the coefficients of linear and quadratic interactions, respectively, *k* is the number of independent parameters, *x_i_* is input predictors or controlling variables (*i* = 1, 2) [44,45]. Therefore, Efficiency % can be expressed as follows:

Efficiency, % = −82.5682 + 41.0017 [pH]–3.1896 [pH]^2^–87.7514 [ACH dose] + 546.7425 [ACH dose]^2^–1.2872 [Time] + 0.0540 [Time]^2^ (4)

Figure 2 depicts observed vs. estimated values of dependent parameters, and Figure 3 presents a bar chart of standardized effects. In the graph, the points form a roughly straight line, which indicates a good fit between the data estimated from the model and the experimental data. A similar relationship was obtained for the removal of malachite green and auramine-O by NaX nanozeolites [46], optimization of lead removal from an aqueous solution by nanocomposite [47], and also for optimization of the removal of ammonium ions from aqueous solutions by pumice as a natural and low-cost adsorbent [48]. In the case of a poor fit, the points would be far away from the straight line [49].

For water treatment, aluminum chlorohydrate (ACH) as a coagulant was used. According to some scientists, ACH is the most concentrated aluminum-based coagulant with the highest basicity [50]. The selected physicochemical parameters of ACH used in this study are shown in Table 10.

Figure 4A,B depicted response surface plots for Efficiency, % with respect to pH and ACH dose (mL/L) for constant Time = 20 min (A), and pH and Time (min) for constant ACH dose = 0.2 mL/L (B). On the one hand, the analysis of the graphs shows that under the established conditions, the highest TOC removal efficiency is achieved at a pH of about 6.5 and using the maximum ACH doses (0.30–0.35 mL/L) and coagulation time (30–35 min). On the other hand, the same conclusions can be drawn in the case of the analysis of graph 4C for the constant pH = 7.0. On this assumption, the highest efficiency is also obtained for the maximum ACH doses (0.30–0.35 mL/L) and coagulation time (30–35 min).

ACH is the water solution of aluminum pentachloride with the general formula Al_2_(OH)_5_Cl∙nH_2_O. ACH added to water undergoes hydrolysis like other soluble aluminum salts used as coagulants, i.e., polyaluminum chloride (PAC), alum (Al_2_(SO_4_)_3_∙14H_2_O), and aluminum chloride (AlCl_3_∙6H_2_O), etc.

Al_2_(OH)_5_Cl + H_2_O → 2Al(OH)_3_↓ + H^+^ + Cl^−^ (5)

As a result of hydrolysis, soluble monomers and polymers are formed, as well as precipitates like Al(OH)_3_. The hydrolysis processes are related to the reduction of alkalinity and pH of the treated water, which depends on the pH of the coagulant solution as well as the aluminum dose applied [51]. The studies carried out so far have shown that the lowest solubility value of Al(OH)_3_ depends on the type of aluminum salt used and is about 6 for alum and about 6.2–6.4 for PACl. The use of aluminum coagulants under these conditions produces the highest amount of Al(OH)_3_ and, at the same time, the lowest concentration of residual aluminum in the treated water. Consequently, the number of removed impurities should be the highest. Studies by other authors have shown that the use of aluminum coagulants is possible at a minimum pH of 5.5–5.8, and additionally, the temperature of the water and the content of other ions in water, such as sulfate (SO_4_^2^), phosphate (PO_4_^3−^), etc., should be taken into account [51,52]. However, in the case of the application of alum for coagulation of grey wastewater, initial pH is of prime importance because the solubility of aluminum compounds strongly depends on pH. In the case of PACl, these dependencies are not strong,; therefore, they can be used in the pH range of 4.5–9.5 [53]. In addition, optimum alum dose increases with an increase in pH in the pH range of 4.5–7.0 [54]. In the other study, a similar observation in the pH range of 5.5–8.4 was described [55].

Aluminum coagulants form soluble monomeric and polymeric molecules and precipitates as follows: Al^3+^, Al(OH)^2+^, Al(OH)_2_^+^, Al(OH)_3_↓, Al(OH)_4_^−^, Al_2_(OH)_2_^4+^, Al_3_(OH)_4_^5+^, and Al_13_O_4_(OH)_24_^7+^. The solid phase particles, i.e., AL(OH)_3,_ contain amphoteric hydroxyl groups that can be positively or negatively charged. The type of charge strongly depends on the pH value. In addition, PACl-type coagulants contain highly charged polymeric forms of aluminum than alum. Al_13_^7+^ was found to be the most common polymeric form. In addition, it is the most stable form over a wider pH range [51,52,53,54,55,56]. Similarly, in the case of ACH, the existence of polymer structures was demonstrated. The Al_13_ polymer, Al_13_O_4_(OH)_24_(H_2_O)_12_Cl_7_, is thought to be a major component of the aluminum chlorohydrate (ACH) polymer system. Al_13_ entity is roughly spherical, with a central tetrahedral Al^3+^ ion surrounded by four shells consisting of O, OH, and H_2_O groups [57]. Nonetheless, the structures of aluminum chlorohydrate monomer, dimer, trimer, and hexamer species were described [58]. Therefore, similar to the PACl-type coagulants, the removal of contaminants is possible thanks to flocs formed by charge naturalization and sweep flocculation [59,60]. Other research findings showed that destabilization of the particles occurs through charge neutralization by adsorption of hydroxide precipitates [61].

Based on the analysis of graphs 4A and 4B, the optimal coagulation pH was assumed to be 6.5, with the dose ACH = 0.35 mL/L. The experimental verification of the model was carried out with a 15-, 20-, 25-, and 30-min coagulation time. The predicted and experimental values of the dependent variable are presented in Table 8. Under these conditions, the highest TOC removal efficiency was achieved after 30 min coagulation time (88.7%). Extending the coagulation time did not increase the efficiency of TOC removal. Predicted and observed (experimental) values were comparable (93.8% vs. 88.7%). Therefore, the following optimal conditions for the coagulation process were assumed: pH = 6.5, ACH dose = 0.35 mL/L, Time 30 min.

Table 9 presents the selected physicochemical parameters of spiked river water after RSM application. Under these conditions, turbidity, color, TSS, COD and TOC decreased by 96.2%, 100%, 97.8%, 70% and 88.7%, respectively. With optimal conditions for the coagulation process of spiked surface water (pH = 6.5 ± 0.1, ACH dose = 0.35 mL/L, Time = 30 min; R^2^ = 0.8799, R^2^_adj_ = 0.7998), the concentrations of TOC, ERY, FLX, AMO, COL, EE, and DIC decreased by 88.7%, 36.4%, 24.7%, 29.0%, 25.5%, 35.4%, 30.4%, respectively. Other studies indicated that the removal rate for diclofenac was 77% [22]. The use of a bench-scale system resulted in the removal of estradiol by 6.9–12.0% [24]. In other studies for DIC, a maximum removal rate of 46% was obtained [25]. Based on published data, the removal rate for ERY, FLX, EE, and DIC was 33%, 15%, 21%, and 8–77%, respectively [22,26,27,28].

Additionally, it should be noted that the total effectiveness of pollutant removal may depend not only on the action mechanisms of coagulants and the process conditions but also on the presence of suspensions in treated water (90 ± 9 mg/L). The suspended particles may support the coagulation and flocculation processes, act as adsorbents, and contribute to the overall efficiency of the removal process. It is clear that the characteristics of the suspended particles in water also affect the performance of coagulation and flocculation processes [62].

## 4. Materials and Methods

### 4.1. Reagents and Chemicals

Ultrapure pharmaceutical standards of DIC, FLX, EE, ERY, AMO, and COL, were purchased from Sigma-Aldrich (St Louis, MO, USA). Acetonitrile for LC-MS, water for LC-MS, and acetic acid ReagentPlus^®^, ≥99%, were purchased from Merck (Darmstadt, Germany). The selected properties of the tested pharmaceuticals are summarized in Table 10.

For pharmaceutical removal studies, all chemicals were at least analytical grade. In addition, deionized water (<1 µS/cm) was used for the preparation of chemicals and dilution of water samples. For pH adjustment, 15% solutions of NaOH or H_2_SO_4_ were used (Chempur, Piekary Śląskie, Poland). Technical grade aluminum chlorohydrate (ACH) was used in this study (Kemipol SA, Police, Poland). Presented in Table 11, the selected physicochemical parameters of the applied coagulant are based on the supplier’s certificate and conducted research.

### 4.2. Sampling Methodology and Spiked River Water

**Figure 5:**
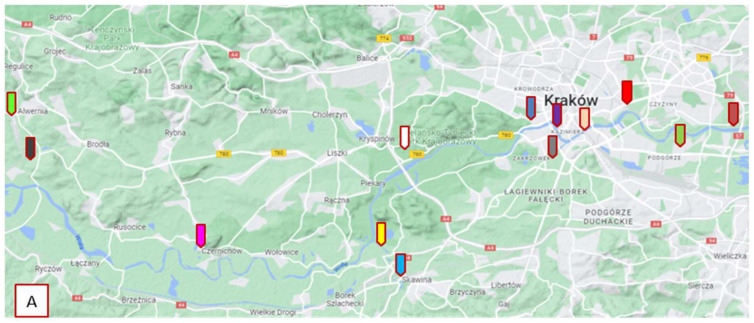

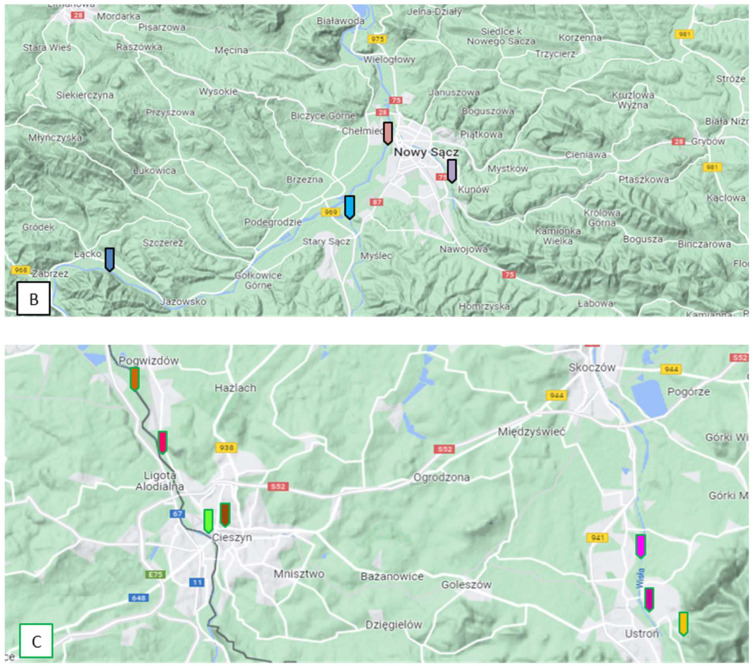
Location of sampling points: **A**—Kraków area; **B**—Nowy Sącz area; **C**—Cieszyn area.

Sampling was carried out in accordance with PN-EN ISO 5667–6: 2016–12 [64] using a bucket with an extension arm. Each sample was taken from places where the water was well mixed. In the case of deep rivers, samples were taken from a depth of about 30 cm below the water table. From rivers whose depth does not exceed 50 cm, extraction was carried out at about 1/3 of the river depth. The sampled bottles were placed in thermal bags at a temperature not exceeding 8 °C and delivered to the laboratory. Analytes extraction was made on the day of sample collection, and until analysis, the extracts were stored at approximately 4 °C. Table 12 presents the description of the samples. The sampling sites are marked on the map in Figure 5.

The spiked water sample was prepared as follows: 10 µg/L of the determined pharmaceuticals (dissolved in a small amount of methanol using ultrasound) were added to the watermarked No. 7, taken from the Vistula River in the center of Krakow, in order to evaluate the suitability of the aluminum chlorohydrate coagulation process to remove pharmaceutical residues.

### 4.3. Analytical Methods

Solid phase extraction (SPE) was used to isolate and concentrate the analytes. 100 mL of each water sample was extracted in a vacuum chamber for the SPE. (Chromabond, MACHEREY-NAGEL, Dueren, Germany). Oasis HLB columns were purchased from Waters (Etten-Leur, The Netherlands). The columns are packed with polymer consisting of two monomer components: lyophilic divinylbenzene and hydrophilic *N*-vinylpyrrolidone. It is a sorbent commonly used to extract compounds with a pH ranging from 1 to 14, showing strong retention of reversed phases.

The determination of pharmaceuticals was performed by reversed-phase liquid chromatography (Nexera, Shimadzu, Kioto, Japan) coupled with tandem mass spectrometry equipped with an electrospray ionization interface (QTrap 3200, AB Sciex, MA, USA). The separation of analytes was performed on a Kinetex 100 × 2.1 mm × 2.6 µm XB C18 100A chromatography column (Phenomenex, CA, USA) with a C18 pre-column (Phenomenex SecurityGuard, CA, USA). The mobile phase was a gradient mixture of 0,1% CH_3_COOH in water and 0,1% CH_3_COOH in acetonitrile. Detection was carried out in the multiple reaction monitoring (MRM) mode. MS/MS parameters for the tested pharmaceuticals are summarised in Table 13.

The determination of pH-value, specific electrical conductivity (SEC), and salinity were performed electrometrically (CPC-401, Elmetron, Poland) according to PN-EN ISO 10523:2012 [65] and PN-EN 27888:1999 [66], respectively. Turbidity and color were performed using a nephelometer (Cyberscan, TBD IR 1000, Eutech Instruments Pte Ltd., Singapore) and spectrophotometer (PF-11, Macherey-Nagel GmbH, Germany) according to PN-EN ISO 7027-1:2016-09 and PN-EN ISO 7887:2012, respectively [67,68]. Gravimetric methods described in PN-EN 872:2007 and PN-ISO 9280:2002 were used for the evaluation of Total Suspended Solids (TSS) and sulfate, respectively [69,70]. The concentration of chloride was determined titrimetrically by Mohr’s method according to PN-ISO 9297:1994 [71]. Chemical Oxygen Demand (COD), Total Organic Carbon (TOC), Total P (TP), Total Nitrogen (TN), and Ammonium Nitrogen (N-NH_4_) were determined spectrophotometrically (PF-11 and NANOCOLOR 500D spectrophotometer) by test kits (Macherey-Nagel, Dueren, Germany).

### 4.4. Design of Experiments (Central Composite Design, CCD and Response Surface Methodology, RSM)

In order to optimize the TOC removal from spiked river water, CCD/RSM was used for three independent parameters, i.e., pH, ACH dose (mL/L), and Time (min). On the basis of some preliminary experiments and literature data, initial ranges for pH, ACH dose, and Time were adopted. TOC was chosen as a dependent parameter because it is one of the main parameters that allows the determination of organic contaminants in water. On the basis of the TOC concentration before and after coagulation, the TOC removal efficiency (%) was calculated. The following parameters were assumed to be constant: temperature (22 ± 2 °C), stirring speed (initially 500 RPM, 1 min, subsequently 100 RPM for the established time), and volume of the treated water (500 mL in each experiment). The planning of the experiments was carried out using the CCD and Statistica 13.3.0 (TIBCO Software Inc., Palo Alto, CA 94304, USA). The implementation of CCD made it possible to obtain the experimental plan presented in Table 3. The plan consisted of 16 experiments (2 experiments in the center of the plan) and was the combination of the set values of three independent parameters (pH, ACH dose, mL/L, and Time, min).

### 4.5. Experimental Study

The experiments were performed with the use of 600 mL beakers and magnetic stirrers with adjustable mixing speeds (Magnetic Stirrer 06-MS-PB, Chemland, Poland). In each experiment, 500 mL of spiked river water was measured into the beaker, the fixed amount of ACH dose was added, and the pH was adjusted to the pH value specified, shown in Table 3. The adjustment of the pH value was performed at 500 RPM for a maximum time of 1 min. The correction of the pH value of treated water was performed with a 15% solution of NaOH or H_2_SO_4_. Thereafter, treated water was stirred for the established time. After a set time, the agitation was turned off, and the samples were left for 30 min for the sedimentation of the precipitated sludge. The determination of turbidity and TSS were performed using unfiltered samples. In the case of other parameters, the treated water was filtered through a 0.45 μm syringe filter. In addition, before RP-HPLC-ESI-MS/MS analysis, the water samples were filtered through a 0.22 μm syringe filter. On the basis of the determined concentration of TOC, it concluded the efficiency of TOC removal in individual experiments. The obtained results of the experiments were analyzed using Statistica 13 to determine how the independent parameters influence the changes of the dependent parameter (Efficiency, %). The results were evaluated statistically, and dependencies between parameters were depicted and presented in 2D graphs. Experimental verification of the model was also carried out in order to check whether the values of the dependent variable estimated from the model are consistent with the experimental values.

## 5. Conclusions

The analysis of twenty-four watercourse samples confirmed that residues of pharmaceuticals such as DIC, FLX, EE, ERY, AMO, and COL are present in samples taken from the Vistula and Olza rivers and their tributaries. It is therefore highly advisable to search for effective methods to remove these compounds from water. This is what future research should focus on. The present study found that the concentration ranges of ERY, FLX, AMO, COL, EE, and DIC in analyzed water samples were 7.58–412.32, 1.21–72.52, 1.22–68.55, 1.28–32.01, 5.36–45.56, 2.20–182.22 ng/L, respectively. In optimal conditions of the coagulation process of spiked surface water, the concentration of TOC, ERY, FLX, AMO, COL, EE, and DIC was decreased by 88.7%, 36.4%, 24.7%, 29.0%, 25.5%, 35.4%, 30.4%, respectively. Simultaneously, turbidity, color, TSS, Total N and N-NH_4_ were decreased by 96.2%, >98.0%, 97.8%, 70.0%, 88.7%, 37.5%, respectively. The study suggests that the effectiveness of the removal of pollutants depends not only on the action mechanisms of coagulants and the process conditions but also on the presence of other substances in the water, including suspensions. These indicated that ACH may be an optional reagent to remove studied pharmaceuticals from contaminated water.

## Figures and Tables

**Table 1:** Concentrations of diclofenac (DIC), fluoxetine (FLX), ethynylestradiol (EE), erythromycin (ERY), amoxicillin(AMO), and colistin (COL) in the tested river samples.

No.	Watercourse Name	Concentration ± SD, ng/L					
		DIC	FLX	EE	ERY	AMO	COL
1	Prądnik	75.07 ± 3.75	5.25 ± 0.20	13.90 ± 0.46	132.50 ± 4.69	23.62 ± 0.84	4.25 ± 0.15
2	Wilga	106.33 ± 3.72	<LOQ *	10.77 ± 0.41	55.32 ± 2.10	16.42 ± 0.48	6.24 ± 0.21
3	Rudawa	105.70 ± 4.02	13.06 ± 0.43	14.90 ± 0.43	412.32 ± 11.96	32.31 ± 1.14	12.54 ± 0.63
4	Sanka	97.67 ± 2.83	<LOQ	12.54 ± 0.48	23.40 ± 0.77	3.56 ± 0.19	19.01 ± 0.67
5	Pychowicki	118.00 ± 4.18	1.58 ± 0.08	14.63 ± 0.42	<LOQ	4.65 ± 0.18	10.21 ± 0.34
6	Dłubnia	75.55 ± 2.12	12.55 ± 0.41	<LOQ	<LOQ	10.40 ± 0.30	1.35 ± 0.05
7	Wisła (Vistula)	27.05 ± 0.89	12.26 ± 0.64	17.56 ± 0.51	12.50 ± 0.41	15.65 ± 0.59	<LOQ
8	Wisła (Vistula)	45.50 ± 1.91	6.22 ± 0.18	8.26 ± 0.43	42.60 ± 1.41	32.11 ± 1.61	1.28 ± 0.04
9	Skawinka	122.67 ± 3.07	22.00 ± 0.78	19.27 ± 1.00	56.42 ± 1.41	23.67 ± 0.83	7.56 ± 0.27
10	Łączany-Skawina	138.10 ± 4.56	45.24 ± 2.35	27.55 ± 0.69	7.88 ± 0.26	8.50 ± 0.43	25.55 ± 1.33
11	Rudno	38.00 ± 1.19	<LOQ	32.75 ± 1.08	<LOQ	<LOQ	2.14 ± 0.08
12	Regulanka	125.27 ± 3.51	2.59 ± 0.09	17.77 ± 0.89	13.40 ± 0.38	35.70 ± 1.26	8.21 ± 0.27
13	Regulanka	100.33 ± 2.51	<LOQ	10.87 ± 0.54	<LOQ	<LOQ	3.01 ± 0.11
14	Dunajec	175.36 ± 5.79	12.45 ± 0.31	12.47 ± 0.44	68.03 ± 2.86	45.55 ± 1.28	26.50 ± 1.11
15	Dunajec	182.22 ± 5.10	16.48 ± 0.54	32.04 ± 1.35	184.22 ± 9.58	29.50 ± 0.97	4.02 ± 0.20
16	Kamienica	12.60 ± 0.42	4.25 ± 0.21	12.54 ± 0.53	92.15 ± 4.61	12.54 ± 0.53	7.12 ± 0.27
17	Poprad	116.21 ± 4.88	22.02 ± 0.92	45.56 ± 1.14	245.51 ± 6.87	16.08 ± 0.80	16.55 ± 0.41
18	Olza	12.56 ± 0.53	4.65 ± 0.23	12.59 ± 0.42	9.63 ± 0.24	35.66 ± 1.78	12.51 ± 0.44
19	Bobrówka	7.26 ± 0.24	2.36 ± 0.06	11.89 ± 0.39	<LOQ	68.55 ± 1.92	32.01 ± 1.60
20	Dobka	2.56 ± 0.13	<LOQ	5.68 ± 0.20	<LOQ	1.22 ± 0.03	7.76 ± 0.40
21	Wisła (Vistula)	2.20 ± 0.06	1.21 ± 0.05	5.36 ± 0.28	7.58 ± 0.27	4.25 ± 0.22	15.61 ± 0.76
22	Olza	12.58 ± 0.42	5.65 ± 0.24	<LOQ	8.88 ± 0.31	12.45 ± 0.62	4.65 ± 0.16
23	Olza	7.96 ± 0.33	72.52 ± 3.77	18.50 ± 0.93	100.05 ± 5.20	7.02 ± 0.23	<LOQ
24	Wisła (Vistula)	2.59 ± 0.11	5.22 ± 0.22	<LOQ	<LOQ	6.25 ± 0.31	<LOQ

* LOQ—Limit of Quantification.

**Table 2:** Selected physicochemical parameters of the spiked river water.

Parameter	Unit	Result *
pH	-	7.7 ± 0.1
Specific Electrical Conductivity (SEC)	µS/cm	5060 ± 506
Salinity	mg NaCl/L	2660 ± 266
Turbidity	NTU	26 ± 2
Color	mg Pt/L	29 ± 3
Total Suspended Solids (TSS)	mg/L	90 ± 9
Chemical Oxygen Demand (COD)	mg O_2_/L	676 ± 101
Total Organic Carbon (TOC)	mg/L	151 ± 23
Chloride	mg/L	1420 ± 213
Sulphate	mg/L	172 ± 26
Total Phosphorus (Total P)	mg/L	<0.3
Total Nitrogen (Total N)	mg/L	3.7 ± 0.4
Ammonium-Nitrogen (N-NH_4_)	mg/L	0.08 ± 0.01
Diclofenac (DIC)	µg/L	9.90 ± 0.61
Fluoxetine (FLX)	µg/L	10.06 ± 0.63
Ethynylestradiol (EE)	µg/L	9.99 ± 0.61
Erythromycin (ERY)	µg/L	10.81 ± 0.67
Amoxicillin (AMO)	µg/L	10.22 ± 0.60
Colistin (COL)	µg/L	9.87 ± 0.64

* parameter value ± the measurement uncertainty for an extension factor k = 2.

**Table 3:** Experimental conditions for the RSM and results (TOC, mg/L, Efficiency, %) for the spiked river water.

Run	Experimental Conditions			Experimental Results *	
	pH	ACH (mL/L)	Time (min)	TOC (mg/L)	Efficiency (%)
1	5.0	0.10	10	87.1 ± 8.7	42.3
2	5.0	0.10	30	69.4 ± 6.9	54.0
3	5.0	0.30	10	42.2 ± 4.2	72.2
4	5.0	0.30	30	26.9 ± 3.0	82.2
5	9.0	0.10	10	125.6 ± 12.6	16.8
6	9.0	0.10	30	85.0 ± 8.5	43.7
7	9.0	0.30	10	91.1 ± 9.1	39.7
8	9.0	0.30	30	71.0 ± 7.1	53.0
9	3.6	0.20	20	140.2 ± 14.0	7.2
10	10.4	0.20	20	142.3 ± 14.2	5.8
11	7.0	0.03	20	103.1 ± 10.3	31.7
12	7.0	0.37	20	23.6 ± 2.4	84.4
13	7.0	0.20	3	89.2 ± 8.9	40.9
14	7.0	0.20	37	38.1 ± 3.81	74.8
15 (C) **	7.0	0.20	20	75.9 ± 7.6	49.7
16 (C)	7.0	0.20	20	77.1 ± 7.1	48.9

* parameter value ± the measurement uncertainty for an extension factor k = 2, ** (C)—center of plan.

**Table 4:** Analysis of the experimental results: evaluation of the effects.

Parameter	Evaluation of Effects, Efficiency, %, R^2^ = 0.8977, R^2^_adj_ = 0.7442, 3 Parameter, 1 Block, 16 Experiments, MS = 148.1306								
	Effect	Standard Error	*p*-Value *	−95% Confidence Interval	+95% Confidence Interval	Factor	Standard Error of Factor	Lower Confidence Interval	Upper Confidence Nterval
Constant value	48.317	8.581	0.0013	27.320	69.313	48.317	8.581	27.320	69.313
pH (L) *	−14.609	6.587	0.0684	−30.726	1.509	−7.304	3.293	−15.363	0.754
pH (Q) **	−25.517	7.997	0.0188	−45.085	−5.948	−12.758	3.999	−22.543	−2.974
ACH dose (L)	26.189	6.587	0.0073	10.072	42.307	13.095	3.293	5.036	21.153
ACH dose (Q)	10.935	7.997	0.2205	−8.634	30.504	5.467	3.999	−4.317	15.252
Time (L)	17.429	6.587	0.0382	1.312	33.546	8.715	3.293	0.656	16.773
Time (Q)	10.793	7.997	0.2258	−8.776	30.362	5.397	3.999	−4.388	15.181
*** pH (L) relative to ACH dose (L)	−6.450	8.606	0.4819	−27.508	14.608	−3.225	4.303	−13.754	7.304
*** pH (L) relative to Time (L)	4.600	8.606	0.6122	−16.458	25.658	2.300	4.303	−8.229	12.829
*** ACH dose (L) relative to Time (L)	−3.800	8.606	0.6742	−24.858	17.258	−1.900	4.303	−12.429	8.629

* statistically significant if *p* < 0.05, * L—linear effect, ** Q—quadratic effect, *** (L) relative to (L)—linear combination of the parameters.

**Table 5:** Analysis of the experiment results after eliminating statistically insignificant linear interactions ((L) relative to (L)) of the parameters–evaluation of the effects.

Parameter	Evaluation of Effects, Efficiency, %, R^2^ = 0.8799, R^2^_adj_ = 0.7998, 3 Parameter, 1 Block, 16 Experiments, MS = 115.9099								
	Effect	Standard Error	*p*-Value *	−95% Confidence Interval	+95% Confidence Interval	Factor	Standard Error of Factor	Lower Confidence Interval	Upper Confidence Interval
Constant value	48.317	7.591	0.0001	31.146	65.488	48.317	7.591	31.146	65.488
pH (L) *	−14.609	5.827	0.0335	−27.789	−1.428	−7.304	2.913	−13.895	−0.714
pH (Q) **	−25.517	7.074	0.0057	−41.520	−9.513	−12.758	3.537	−20.760	−4.757
ACH dose (L)	26.189	5.827	0.0015	13.008	39.370	13.095	2.913	6.504	19.685
ACH dose (Q)	10.935	7.074	0.1566	−5.068	26.938	5.467	3.537	−2.534	13.469
Time (L)	17.429	5.827	0.0152	4.248	30.610	8.715	2.913	2.124	15.305
Time (Q)	10.793	7.074	0.1614	−5.210	26.797	5.397	3.537	−2.605	13.398

* statistically significant if *p* < 0.05, * L—linear effect, ** Q—quadratic effect.

**Table 6:** Analysis of the experimental results: verification of the adequacy of the model by ANOVA.

Parameter	Evaluation of Effects, Efficiency, %, R^2^ = 0.8799, R^2^_adj_ = 0.7998, 3 Parameter, 1 Block, 16 Experiments, MS = 115.9099			
	SS ***	**** MS	***** F	*p*-Value *
pH (L) *	728.642	728.642	6.286	0.0330
pH (Q) **	1507.951	1507.951	13.010	0.0057
ACH dose (L)	2341.706	2341.706	20.203	0.0015
ACH dose (Q)	276.930	276.930	2.389	0.1566
Time (L)	1037.138	1037.138	8.948	0.0152
Time (Q)	269.814	269.814	2.328	0.1614
Error	1043.189	115.910	-	-

* L—linear effect, ** Q—quadratic effect, *** SS—predicted residual error of sum of squares, **** MS—mean square error, ***** F statistics.

**Table 7:** Calculated linear (L) and quadratic (Q) coefficients of the fitted model.

Parameter		Regression Coefficients, R^2^ = 0.8799, R^2^_adj_ = 0.7998, 3 Parameter, 1 Block, 16 Experiments, MS = 115.9099				
	Regression Coefficient	*** SE	*t*-Value **** df = 9	95% Confidence Interval Lower Limit	95% Confidence Interval Upper Limit	***** *p*-Value *
Intercept	−82.5682	53.9597	−1.5302	−204.634	39.497	0.1603
pH (L) *	41.0017	12.4655	3.2892	12.803	69.201	0.0094
pH (Q) **	−3.1896	0.8843	−3.6069	-5.190	−1.189	0.0057
ACH dose (L)	−87.7514	144.4556	−0.6075	−414.533	239.030	0.5586
ACH dose (Q)	546.7425	353.7185	1.5457	−253.424	1346.909	0.1566
Time (L)	−1.2872	1.4446	−0.8911	−4.555	1.981	0.3961
Time (Q)	0.0540	0..354	1.5257	−0.026	0.134	0.1614

* L—linear effect, ** Q—quadratic effect, *** SE—standard error, **** df—degree of freedom, ***** statistically significant if *p* < 0.05.

**Table 8:** TOC of spiked river water after RSM application (optimal conditions, pH = 6.5 ± 0.1, ACH dose = 0.35 mL/L, Time = 15, 20, 25, and 30 min)—experimental model verification.

Parameter	Effect, % After 15 min	Effect, % After 20 min	Effect, % After 25 min	Effect, % After 30 min
Total Organic Carbon, predicted	81.2	82.5	86.5	93.5
Total Organic Carbon, experimental	77.5	78.5	81.0	88.7

**Table 9:** Selected physicochemical parameters of spiked river water after RSM application (optimal conditions, pH = 6.5 ± 0.1, ACH dose = 0.35 mL/L, Time = 30 min).

Parameter	Unit	Result *	Effect (%) **
pH	-	6.5 ± 0.1	↓ 25.3
Specific Electrical Conductivity (SEC)	µS/cm	5350 ± 535	↑ 5.7
Salinity	mg NaCl/L	2820 ± 282	↑ 6.0
Turbidity	NTU	1.0 ± 0.1	↓ 96.2
Color	mg Pt/L	<2	↓ >98.0
Total Suspended Solids (TSS)	mg/L	2.0 ± 0.1	↓ 97.8
Chemical Oxygen Demand	mg O_2_/L	203 ± 30	↓ 70.0
Total Organic Carbon	mg/L	17.0 ± 2.6	↓ 88.7
Chloride	mg/L	1525 ± 229	↑ 10.6
Sulphate	mg/L	160 ± 24	↓ 7.0
Total Phosphorus (Total P)	mg/L	<0.3	Not significant
Total Nitrogen (Total N)	mg/L	1.7 ± 0.2	↓ 88.7
Ammonium-Nitrogen (N-NH_4_)	mg/L	0.05 ± 0.01	↓ 37.5
Diclofenac	µg/L	6.89 ± 0.43	↓ 30.4
Fluoxetine	µg/L	7.58 ± 0.49	↓ 24.7
Ethynylestradiol	µg/L	6.45 ± 0.42	↓ 35.4
Erythromycin	µg/L	6.88 ± 0.41	↓ 36.4
Amoxicillin	µg/L	7.26 ± 0.44	↓ 29.0
Colistin	µg/L	7.35 ± 0.43	↓ 25.5

* parameter value ± the measurement uncertainty for an extension factor k = 2, ** Effect = $\frac{\left(C1-C2\right)\times 100\%}{C1}$ and ** Effect = $\frac{\left(C2-C1\right)\times 100\%}{C1}\;$(for SEC, Salinity, chloride), where c_1_-concentration in spiked river water, c_2_-concentration in treated river water, ↑–increase in the parameter value, ↓–decrease in the parameter value.

**Table 10:** Selected properties of the tested pharmaceuticals [63].

Name (Shortcut)	Formula, Molar Mass, g/mol	CAS No.	Type	Action
Diclofenac (DIC)	C_14_H_11_C_l2_NO_2_, 296.15	15307-86-5 15307-79-6 (sodium salt) 15307-81-0 (potassium salt)	non-steroidal anti-inflammatory drug	anti-inflammatory, analgesic. antipyretic
Fluoxetine (FLX)	C_17_H_18_F_3_NO, 309.30	54910-89-3	antidepressant drug	treatment of depressive and obsessive -compulsive disorders
Ethinylestradiol (EE)	C_2_0H_24_O_2_, 296.40	57-63-6	synthetic estrogen	component of two-component contraceptives
Erythromycin (ERY)	C_37_H_67_NO_13_, 733.93	114-07-8	an antibiotic from the group of macrolides	infections of the upper and lower respiratory tract
Amoxicillin (AMO)	C_16_H_19_N_3_O_5_S, 365.40	26787-78-0 34642-77-8 (sodium salt) 34642-78-9 (potassium salt)	a semi-synthetic β-lactam antibiotic	gastrointestinal and urinary infections, upper and lower respiratory tract infections
Colistin (COL)	C_52_H_98_N_16_O_13_ 1155.45	1264-72-8	an antibiotic belonging to polymyxins	urinary tract infections, mainly used in veterinary medicine

**Table 11:** Selected physicochemical parameters of the aluminum chlorohydrate (ACH).

Parameter	Unit	Result
pH	-	3.5 ± 0.1
Appearance	-	clear
Aluminum	%	12.4
Aluminum (as Al_2_O_3_)	%	23.5
Basicity	%	82.5
Total Iron (as Fe)	mg/L	24

**Table 12:** A detailed description of the water samples.

No.	Watercourse Name	Location		Marker
1	Prądnik	Krakow	in the Dąbie district	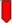
2	Wilga	Krakow	in the Ludwinów district	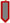
3	Rudawa	Krakow	in the Salwator district	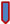
4	Sanka	Krakow	in the Bielany district	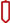
5	Pychowicki	Krakow	in the Podgórze district	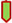
6	Dłubnia	Krakow	in the Mogiła district	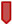
7	Wisła (Vistula)	Krakow	in the city center	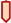
8	Wisła (Vistula)	Krakow	in the city center	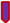
9	Skawinka	Skawinka	in the city center	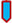
10	Łączany-Skawina	Kopanka	in a village located near the Krakow	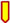
11	Rudno	Czernichów	in a village located near the Krakow	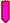
12	Regulanka	Alwernia	in the city center	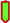
13	Regulanka	Okleśna	in a village located near the Alwernia	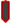
14	Dunajec	Nowy Sącz	in the city center	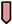
15	Dunajec	Łącko	in a village located near the Nowy Sącz	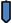
16	Kamienica	Nowy Sącz	in the suburbs	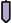
17	Poprad	Nowy Sącz	in the city center	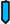
18	Olza	Cieszyn	border crossing between Poland and the Czech Republic	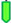
19	Bobrówka	Cieszyn	tributary to the Olza river	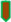
20	Dobka	Ustroń	the source of a mountain stream	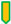
21	Wisła (Vistula)	Ustroń	for municipal consumption	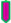
22	Olza	Pogwizdów	a small village with a small population	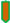
23	Olza	Marklowice	at the sewage treatment plant	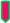
24	Wisła (Vistula)	Hermanice	after the sewage treatment plant	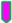

**Table 13:** MS/MS parameters for diclofenac (DIC), fluoxetine (FLX), ethynylestradiol (EE), erythromycin (ERY), amoxicillin (AMO), and colistin (COL).

Analyte	Precursor Ions, *m*/*z*	Fragmentation Ions, *m*/*z*	Collision Energy, eV
DIC	294.000	250.000 214.000	36
FLX	309.700	148.000 44.200	15
EE	295.000	159.000 145.000	15
ERY	734.600	158.400 576.500	39
AMO	366.200	348.800 114.000	16
COL	585.500	341.000 100.800	30

## Data Availability

Not applicable.

## Sample Availability

Samples of the compounds are available from the authors.

## References

[1] European Commission Population Structure and Ageing Eurostat 2022. https://ec.europa.eu/eurostat/statistics-explained/index.php?title=Population_structure_and_ageing.

[2] Bley S.J. (2020). Sustainable Development in the European Union. Monitoring Report on Progress Towards the SDGs in an EU Context.

[3] Klamar A., Kitzmann F., Kirch W. (2012). Pharmaco-economic impact of demographic change on pharmaceutical expenses in Germany and France. BMC Public Health.

[4] (2017). World Health Organization Drinking Water Parameter Cooperation Project. https://ec.europa.eu/environment/water/water-drink/pdf/WHO_parameter_report.pdf.

[5] Lee J., Ji K., Lim Kho Y., Kim P., Choi K. (2011). Chronic exposure to diclofenac on two freshwater cladocerans and Japanese medaka. Ecotoxicol. Environ. Saf..

[6] Mehinto A.C., Hill E.M., Tyler C.R. (2010). Uptake and biological effects of environmentally relevant concentrations of the nonsteroidal anti-inflammatory pharmaceutical diclofenac in rainbow trout (*Oncorhynchus mykiss*). Environ. Sci. Technol..

[7] Nambirajan K., Muralidharan S., Ashimkumar A.R., Jedhav S. (2021). Nimesulide Poisoning in White-Rumped Vulture Gyps Bengalensis in Gujarat. Environ. Sci. Pollut. Res..

[8] Prakash V., Galligan T.H., Chakraborty S.S., Dave R., Kulkarni M.D., Prakash N., Shringarpure R.N., Ranade S.P., Green R.E. (2019). Recent changes in populations of Critically Endangered Gyps vultures in India. Bird Conserv. Int..

[9] García Hernández M.P., Cabas I., Rodenas M.C., Arizcun M., Chaves-Pozo E., Power D.M., García Ayala A. (2020). 17α-ethynylestradiol prevents the natural male-to-female sex change in gilthead seabream (*Sparus aurata* L.). Sci. Rep..

[10] Weinberger J., Klaper R. (2014). Environmental concentrations of the selective serotonin reuptake inhibitor fluoxetine impact specific behaviors involved in reproduction, feeding and predator avoidance in the fish Pimephales promelas (fathead minnow). Aquat. Toxicol..

[11] European Commission Communication from the Commission to the European Parliament, the Council and the European Economic and Social Committee, European Union Strategic Approach to Pharmaceuticals in the Environment. Brussels, 11 March 2019 COM(2019) 128 Final. https://ec.europa.eu/transparency/documents-register/api/files/COM(2019)128_0/de00000000082030?rendition=false.

[12] Chopra S., Kumar D. (2018). Pharmaceuticals and personal care products (PPCPs) as emerging environmental pollutants: Toxicity and risk assessment. Advances in Animal Biotechnology and Its Applications.

[13] (2013). Directive 2013/39/EU of the European Parliament and of the Council of 12 August 2013 Amending Directives 2000/60/EC and 2008/105/EC as Regards Priority Substances in the Field of Water Policy Text with EEA Relevance. Official Journal of the European Union. Brussels. https://eur-lex.europa.eu/LexUriServ/LexUriServ.do?uri=OJ:L:2013:226:0001:0017:EN:PDF.

[14] Esbele A.J., Abou-Elwafa Abdallah M., Harrad S. (2017). Pharmaceuticals and personal care products (PPCPs) in the freshwater aquatic environment. Emerg. Contam..

[15] Liu J.L., Wong M.H. (2013). Pharmaceuticals and personal care products (PPCPs): A re-view on environmental contamination in China. Environ. Int..

[16] Arias J. (2019). Pharmaceutical and Personal Hygiene Products (PPcPs): A Threat Little Studied in Colombian Waters. Agric. Res. Technol..

[17] Arman N.Z., Salmiati S., Aris A., Salim M.R., Nazifa T.H., Muhamad M.S., Marpongahtun M. (2021). A Review on Emerging Pollutants in the Water Environment: Existences, Health Effects and Treatment Processes. Water.

[18] Stefanakis A.I., Becker J.A. (2016). A review of emerging contaminants in water: Classification, sources, and potential risks. In Impact of Water Pollution on Human Health and Environmental Sustainability. IGI Glob..

[19] Grela A., Kuc J., Bajda T. (2021). A Review on the Application of Zeolites and Mesoporous Silica Materials in the Removal of Non-Steroidal Anti-Inflammatory Drugs and Antibiotics from Water. Materials.

[20] Peña-Guzmán C., Ulloa-Sánchez S., Mora K., Helena-Bustos R., Lopez-Barrera E., Alvarez J., Rodriguez-Pinzón M. (2019). Emerging Pollutants in the Urban Water Cycle in Latin America: A Review of the Current Literature. J. Environ. Manag..

[21] Khan N.A., Ahmed S., Vambol V., Vambol S. (2021). Pharmaceutical Wastewater Treatment Technologies: Concepts and Implementation Strategies.

[22] Vieno N., Tuhkanen T., Kronberg L. (2006). Removal of Pharmaceuticals in Drinking Water Treatment: Effect of Chemical Coagulation. Environ. Technol..

[23] Yang W., Wu Y., Zhang L., Jiang J., Feng L. (2014). Removal of five selected pharmaceuticals by coagulation in the presence of dissolved humic acids and kaolin. Desalin. Water Treat..

[24] Bundy M.M., Doucette W.J., McNeill L., Ericson J.F. (2007). Removal of pharmaceuticals and related compounds by a bench-scale drinking water treatment system. J. Water Supply Res. Technol. Aqua.

[25] Suarez S., Lema J.M., Omil F. (2009). Pre-treatment of hospital wastewater by coagulation–flocculation and flotation. Bioresour. Technol..

[26] Vieno N.M., Härkki H. (2007). Occurrence of pharmaceuticals in river water and their elimination in a pilot-scale drinking water treatment plant. Environ. Sci. Technol..

[27] Bodzek M., Dudziak M. (2006). Elimination of steroidal sex hormones by conventional water treatment and membrane processes. Desalination.

[28] Westerhoff P., Yoon Y., Snyder S., Wert E. (2005). Fate of endocrine-disruptor, pharmaceutical, and personal care product chemicals during simulated drinking water treatment processes. Environ. Sci. Technol..

[29] Jonathan T.A., Hai F.I., Al-aboud T.M. (2012). Chemical coagulation-based processes for trace organic contaminant removal: Current state and future potential. J. Environ. Manag..

[30] Drzewicz P., Drobniewska A., Sikorska K., Nałęcz-Jawecki G. (2019). Analytical and ecotoxicological studies on degradation of fluoxetine and fluvoxamine by potassium ferrate. Environ. Technol..

[31] Omar I.A., Aziz S.Q. (2021). Optimization of ACH coagulant, settling time and powdered activated carbon as coagulant aid with economic analysis. Glob. NEST J..

[32] Verma A.K., Bhunia P., Dash R.R. (2012). Effectiveness of Aluminum Chlorohydrate (ACH) for Decolorization of Silk Dyebath Effluents. Ind. Eng. Chem. Res..

[33] Sewage Flows into the Dunajec. “Very Harmful Pollution”. In Oryginal: Ścieki Wpadają do Dunajca. “Bardzo Szkodliwe Zanieczyszczenie”. In Press, Autors: Wini/ks/, 2019. https://tvn24.pl/krakow/malopolska-zanieczyeniem-dunajca-oczyszczalnie-nie-wyrabia-ra958876-2313115.

[34] Baranowska I., Kowalski B. (2012). A rapid UHPLC methodfor the simultaneous determination of drugs from different therapeutic groups in surface water and wastewater. Bull. Environ. Contam. Toxicol..

[35] Giebułtowicz J., Nałęcz-Jawecki G. (2014). Occurrence of antidepressant residues in the sewage-impacted Vistula and Utrata rivers and in tap water in Warsaw (Poland). Ecotoxicol. Environ. Saf..

[36] Savcı S. (2013). A review of occurrence of pharmaceuticals in sediments. Afr. J. Biotechnol..

[37] Kondor A.C., Molnár É., Jakab G., Vancsik A., Filep T., Szeberényi J., Szabó L., Maász G., Pirger Z., Weiperth A. (2022). Pharmaceuticals in water and sediment of small streams under the pressure of urbanization: Concentrations, interactions, and risks. Sci. Total Environ..

[38] Kucharski D., Nałęcz-Jawecki G., Drzewicz P., Skowronek A., Mianowicz K., Strzelecka A., Giebułtowicz J. (2022). The assessment of environmental risk related to the occurrence of pharmaceuticals in bottom sediments of the Odra River estuary (SW Baltic Sea). Sci. Total Environ..

[39] Trinh T.K., Kang L.S. (2010). Application of Response Surface Method as an Experimental Design to Optimize Coagulation Tests. Environ. Eng. Res..

[40] Thomas M., Zdebik D., Białecka B. (2018). Use of sodium trithiocarbonate for remove of chelated copper ions from industrial wastewater originating from the electroless copper plating process. Arch. Environ. Prot..

[41] Thomas M., Zdebik D. (2019). Treatment of Real Textile Wastewater by Using Potassium Ferrate(VI) and Fe(III)/H_2_O_2_. Application of Aliivibrio fischeri and Brachionus plicatilis Tests for Toxicity Assessment. Fibres Text. East. Eur..

[42] Kucharski P., Białecka B., Thomas M. (2022). Removal of cadmium ions from polluted waters using low-cost adsorbents: Process optimization study. Desalin. Water Treat..

[43] Thomas M., Zdebik D., Białecka B. (2018). Using Sodium Trithiocarbonate to Precipitate Heavy Metals from Industrial Wastewater—from the Laboratory to Industrial Scale. Pol. J. Environ. Stud..

[44] Montgomery D.C. (2001). Design and Analysis of Experiments.

[45] Khettaf S., Khouni I., Louhichi G., Ghrabi A., Bousselmi L., Bouhidel K.-E., Bouhelassa M. (2021). Optimization of coagulation–flocculation process in the treatment of surface water for a maximum dissolved organic matter removal using RSM approach. Water Supply.

[46] Shojaei S., Shojaei S., Band S.S., Farizhandi A., Abbas K., Ghoroqi M., Mosavi A. (2021). Application of Taguchi method and response surface methodology into the removal of malachite green and auramine-O by NaX nanozeolites. Sci. Rep..

[47] Javanbakht V., Ghoreishi S.M. (2017). Application of response surface methodology for optimization of lead removal from an aqueous solution by a novel superparamagnetic nanocomposite. Adsorpt. Sci. Technol.

[48] Moradi M., Fazlzadehdavil M., Pirsaheb M., Mansouri Y., Khosravi T., Sharafi K. (2016). Response surface methodology (RSM) and its application for optimization of ammonium ions removal from aqueous solutions by pumice as a natural and low cost adsorbent. Arch. Environ. Prot..

[49] Som A.M., Ramlee A.A., Puasa S.W., Hamid H.A.A. (2021). Optimisation of operating conditions during coagulation-flocculation process in industrial wastewater treatment using Hylocereus undatus foliage through response surface methodology. Environ. Sci. Pollut. Res. Int..

[50] Naceradska J., Pivokonska L., Pivokonsky M. (2019). On the importance of pH Value in Coagulation, Journal of Water Supply: Research and Technology. J. Water Supply Res. Technol..

[51] Pernitsky D.J., Edzwald J.K. (2006). Selection of alum and polyaluminum coagulants: Principles and applications. J. Water Suppl..

[52] Kvech S., Edwards M. (2002). Solubility controls on aluminum in drinking water at relatively low and high pH. Water Res..

[53] Vinitha E.V., Mansoor Ahammed M., Gadekar M.R. (2018). Chemical coagulation of greywater: Modelling using artificial neural networks. Water Sci. Technol..

[54] Pidou M., Avery L., Stephenson T., Jeffrey P., Simon A.P. (2008). Chemical solutions for greywater recycling. Chemosphere.

[55] Ghaitidak D.M., Yadav K.D. (2015). Reuse of greywater: Effect of coagulant. Desalin. Water Treat..

[56] Edzwald J., Haarhoff J. (2011). Dissolved Air Flotation for Water Clarification.

[57] Pophristic V., Balagurusamy V.S.K., Klein M.L. (2004). Structure and dynamics of the aluminum chlorohydrate polymer Al_13_O_4_(OH)_24_(H_2_O)_12_Cl_7_. Phys. Chem. Chem. Phys..

[58] Pophristic V., Klein M.L., Holerca M.N. (2004). Modeling Small Aluminum Chlorohydrate Polymers. J. Phys. Chem. A.

[59] Ghernaout D., Ghernaout B. (2012). Sweep flocculation as a second form of charge neutralization—A review. Desalin. Water Treat..

[60] Bratby J. (2016). Coagulation and Flocculation in Water and Wastewater Treatment.

[61] Pourrezaei P., Drzewicz P., Wang Y., Gamal El-Din M., Perez-Estrada L.A., Martin J.W., Anderson J., Wiseman S., Liber K., Giesy J.P. (2011). The Impact of Metallic Coagulants on the Removal of Organic Compounds from Oil Sands Process-Affected Water. Environ. Sci. Technol..

[62] Kurniawan S.B., Abdullah S.R.S., Imron M.F., Said N.S.M., Ismail N., Hasan H.A., Othman A.R., Purwanti I.F. (2020). Challenges and Opportunities of Biocoagulant/Bioflocculant Application for Drinking Water and Wastewater Treatment and Its Potential for Sludge Recovery. Int. J. Environ. Res. Public Health.

[63] DrugBank Online Database for Drug and Drug Target Info. https://go.drugbank.com/.

[64] (2016). Water Quality—Sampling—Part 6: Guidelines for Sampling Rivers and Streams.

[65] (2008). Water Quality—Determination of pH.

[66] (1999). Water Quality—Determination of Electrical Conductivity.

[67] (2016). Water Quality. Determination of Turbidity.

[68] (2012). Water Quality—Examination and Determination of Colour.

[69] (2007). Water Quality. Determination of Suspended Solids. Method by Filtration through Glass Fibre Filters.

[70] (2002). Water Quality—Determination of sulfate—Gravimetric Method using Barium Chloride.

[71] (1994). Water Quality—Determination of Chloride—Silver Nitrate Titration with Chromate Indicator (Mohr’s Method).

